# Activation of the Small G Protein Arf6 by Dynamin2 through Guanine Nucleotide Exchange Factors in Endocytosis

**DOI:** 10.1038/srep14919

**Published:** 2015-10-27

**Authors:** Risa Okada, Yohei Yamauchi, Tsunaki Hongu, Yuji Funakoshi, Norihiko Ohbayashi, Hiroshi Hasegawa, Yasunori Kanaho

**Affiliations:** 1 Department of Physiological Chemistry, Faculty of Medicine and Graduate School of Comprehensive Human Sciences, University of Tsukuba, 1-1-1 Tennodai, Tsukuba 305-8575, Japan

## Abstract

The small G protein Arf6 and the GTPase dynamin2 (Dyn2) play key roles in clathrin-mediated endocytosis (CME). However, their functional relationship remains obscure. Here, we show that Arf6 functions as a downstream molecule of Dyn2 in CME. Wild type of Dyn2 overexpressed in HeLa cells markedly activates Arf6, while a GTPase-lacking Dyn2 mutant does not. Of the Arf6-specific guanine nucleotide exchange factors, EFA6A, EFA6B, and EFA6D specifically interact with Dyn2. Furthermore, overexpression of dominant negative mutants or knockdown of EFA6B and EFA6D significantly inhibit Dyn2-induced Arf6 activation. Finally, overexpression of the binding region peptide of EFA6B for Dyn2 or knockdown of EFA6B and EFA6D significantly suppresses clathrin-mediated transferrin uptake. These results provide evidence for a novel Arf6 activation mechanism by Dyn2 through EFA6B and EFA6D in CME in a manner dependent upon the GTPase activity of Dyn2.

Endocytosis, by which cells internalize proteins of the plasma membrane with extracellular cargo molecules, controls the signaling output of receptors, mediates cellular uptake of nutrients, and is exploited by pathogens to enter cells^1^. The small G protein ADP-ribosylation factor 6 (Arf6) and the GTPase dynamin (Dyn) play key roles in forming free endocytic vesicles during clathrin-mediated endocytosis (CME)^2^.

Three mammalian Dyn isoforms, Dyn1, 2, and 3, have been identified^3^. Dyn1 is expressed predominantly in neuronal cells, while expression of Dyn2 is ubiquitous^4^,^5^,^6^. Dyn3 is primarily expressed in the testis and the nervous system^7^,^8^. Dyn2 regulates CME by assembling in helical polymers at the neck of budding membranes and promoting scission of the invaginated membrane in a manner dependent on its conformational change induced upon GTP hydrolysis^2^,^9^.

Arf functions as a molecular switch in various signal transduction pathways by cycling between GDP-bound inactive and GTP-bound active forms, which is precisely regulated by the guanine nucleotide exchange factors (GEFs) and GTPase-activating proteins (GAPs)^10^,^11^. Of 6 Arf family members, Arf1-6, which are divided into 3 classes based on their sequence homology^12^, Arf6, the sole member of class III, exclusively locates at the plasma membrane and endosomal compartments to play important roles in membrane dynamics-based cell events through the regulation of actin cytoskeleton reorganization^10^,^13^,^14^.

Although the link between Dyn2 and Arf6 in CME via the NDP kinase NM23-H1 has been previously shown^15^,^16^,^17^, the report suggesting that actin polymerization is involved in vesicle scission in addition to neck elongation and movement of vesicles into the cell during CME^18^,^19^,^20^ led us to speculate that Arf6 might support the membrane scission facilitated by Dyn2 by reorganizing actin cytoskeleton. Here, we investigate the functional relationship between Dyn2 and Arf6 in CME. The results demonstrate that Dyn2 activates Arf6 through its GEFs, EFA6B and EFA6D, in a manner dependent on Dyn2 GTPase activity, providing a novel insight into the molecular mechanism of CME.

## Results

### Dyn2 Activates Arf6

**Figure 1:**
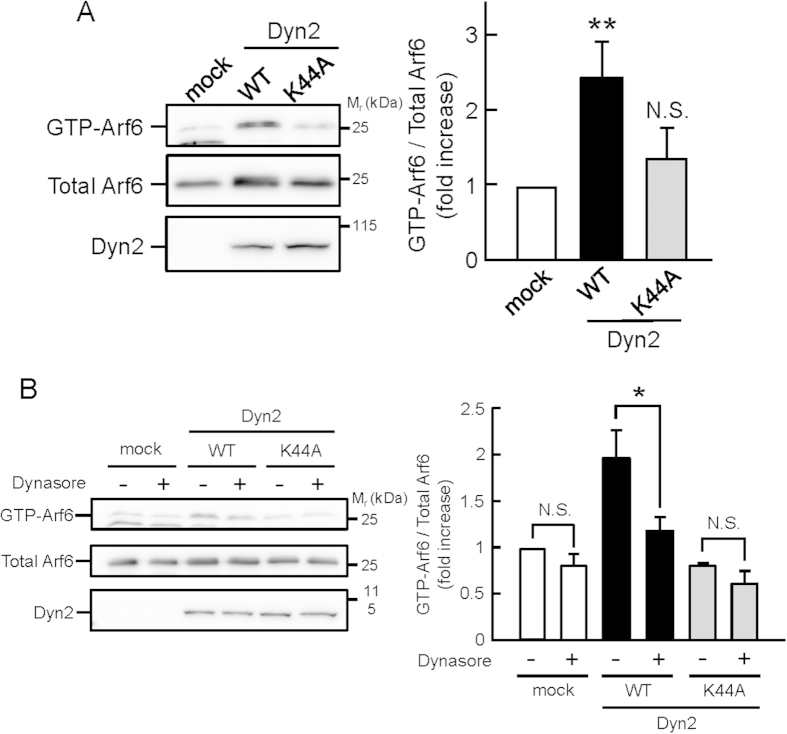
Dyn2 activates Arf6 in a manner dependent on its GTPase activity. (**A**) HA-tagged wild type of Dyn2 or its GTPase-deficient mutant K44A was coexpressed with Arf6-Flag in HeLa cells. After 24 hr, the active GTP-Arf6 was pulled down with glutathione-Sepharose beads conjugated with glutathione *S*-transferase (GST)-tagged leucine zipper region II (LZII) (amino acids 398–455) of JNK-interacting protein (JIP), which specifically binds to the active form of Arf6, and immunoblotted with anti-Flag antibody (left panel). Total Arf6 and Dyn2 expressed in the cell were also immunoblotted with anti-Flag and -HA antibodies, respectively. Right panel shows the means ± SEM of the levels of GTP-Arf6 from eight independent experiments. Statistical significance was calculated using Tukey multiple comparison test; **P < 0.01 and N.S., not significant. (**B**) HeLa cells expressed with wild type of HA-Dyn2 or its K44A mutant and Arf6-Flag were treated with 80 μM dynasore at 37 °C for 30 min, and the levels of GTP-Arf6 and total Arf6 were detected as in (**A**) (left panel). Right panel shows the means ± SEM of the levels of GTP-Arf6 from three independent experiments. Statistical significance was calculated using Tukey multiple comparison test; *P < 0.05 and N.S., not significant.

To investigate the functional relationship between Dyn2 and Arf6, wild type or a GTPase-lacking mutant of Dyn2^21^ (WT or K44A) tagged with HA at its N-terminus was coexpressed with Arf6 tagged with Flag at its C-terminus in HeLa cells. Interestingly, WT Dyn2 markedly activated Arf6, while its GTPase-lacking mutant K44A did not (Fig. 1A), suggesting activation of Arf6 by Dyn2 in a manner dependent on the GTPase activity of Dyn2. Consistent with these results, treatment of cells with dynasore, an inhibitor of the GTPase activity of Dyn^22^, significantly suppressed the Dyn2-induced Arf6 activation without significant effects on the levels of GTP-Arf6 in control and K44A-expressed cells (Fig. 1B).

### Dyn2-Induced Arf6 Activation Is Mediated by EFA6

**Figure 2:**
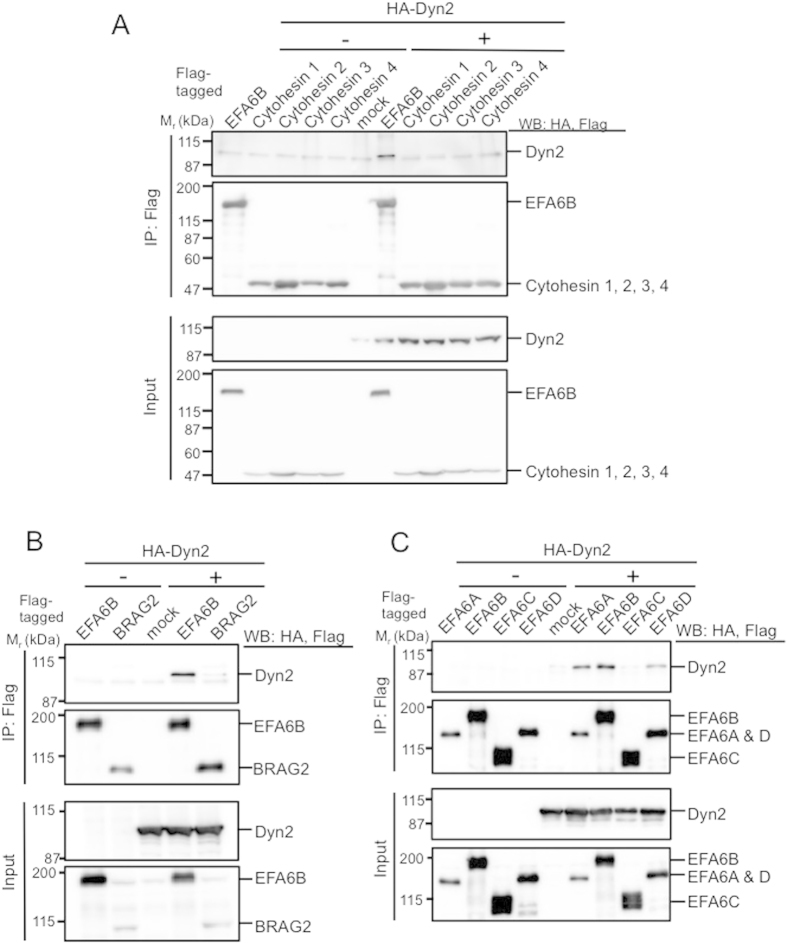
Interaction of EFA6s with Dyn2. Flag-cytohesin isoforms and -EFA6B (**A**), Flag-EFA6B and -BRAG2 (**B**), and Flag-EFA6 isoforms (**C**) were individually coexpressed with HA-Dyn2 in HEK293T cells. After 48 hr of expression, GEFs were immunoprecipitated with anti-Flag antibody beads, and coimmunoprecipitated Dyn2 was assessed by western blotting probed with anti-HA antibody.

As the activation of Arf is tightly regulated by GEFs, we hypothesized that Dyn2-induced Arf6 activation is mediated by an Arf6-specific GEF(s). Of the 15 Arf GEFs identified thus far, 7 GEFs, including cytohesin2–3, EFA6A-D, and BRAG2, are specific to Arf6^23^. To investigate whether an Arf6 GEF is implicated in Dyn2-induced Arf6 activation, we examined the interaction of each GEF with Dyn2 (Fig. 2). In this experiment, Flag-GEFs coexpressed with HA-Dyn2 in HEK293T cells were immunoprecipitated, and coimmunoprecipitated Dyn2 was assessed. Although none of cytohesin family proteins interacted with Dyn2, EFA6B bound to Dyn2 (Fig. 2A). BRAG2 interacted with Dyn2 very little, if any (Fig. 2B). In addition to EFA6B, EFA6A and EFA6D also bound to Dyn2, but EFA6C did not (Fig. 2C). Thus, it is likely that Dyn2 activates Arf6 through EFA6A, EFA6B and/or EFA6D.

**Figure 3:**
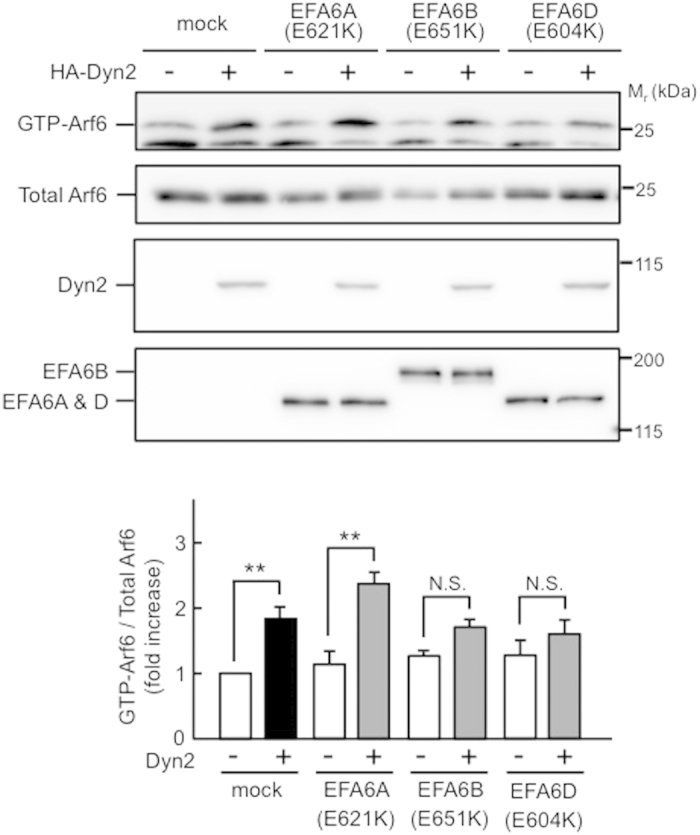
Effects of dominant negative EFA6 mutants on Dyn2-induced Arf6 activation. Flag-tagged dominant negative mutants of EFA6s, EFA6A (E621K), EFA6B (E651K) or EFA6D (E604K), were coexpressed with Arf6-Flag and with or without HA-Dyn2 in HEK293T cells. After 24 hr of expression, the levels of GTP-Arf6 were detected as in Fig. 1A (upper panels). Lower panel represents the mean ± SEM of the levels of GTP-Arf6 from five independent experiments. Statistical significance was calculated using Tukey multiple comparison test; **P < 0.01 and N.S., not significant.

To confirm this notion, the effects of dominant negative mutants of EFA6s on Dyn2-induced Arf6 activation were examined. Expression of the GEF activity-deficient mutants, EFA6B (E651K) and EFA6D (E604K), significantly inhibited Dyn2-induced Arf6 activation (Fig. 3). Although the mutant EFA6A (E621K) interacted with Dyn2 (Supplementary Fig. S1), it did not inhibit the Dyn2-induced Arf6 activation, indicating that the interaction of this mutant is not functional. We also tested the effects of the dominant negative mutant EFA6C (E377K). This mutant markedly reduced the basal level of GTP-Arf6 with unknown reason (data not shown), making it very difficult to correctly assess its effect on the Dyn2-induced Arf6 activation. Furthermore, we examined the effects of knockdown of EFA6B and EFA6D on Dyn2-induced Arf6 activation. Knockdown of EFA6B and EFA6D significantly suppressed the Dyn2-induced Arf6 activation (Supplementary Fig. S2). These results, taken together, conclude that EFA6B and EFA6D mediate Arf6 activation by Dyn2.

### Colocalization of EFA6s with Dyn2 in HeLa cells

**Figure 4:**
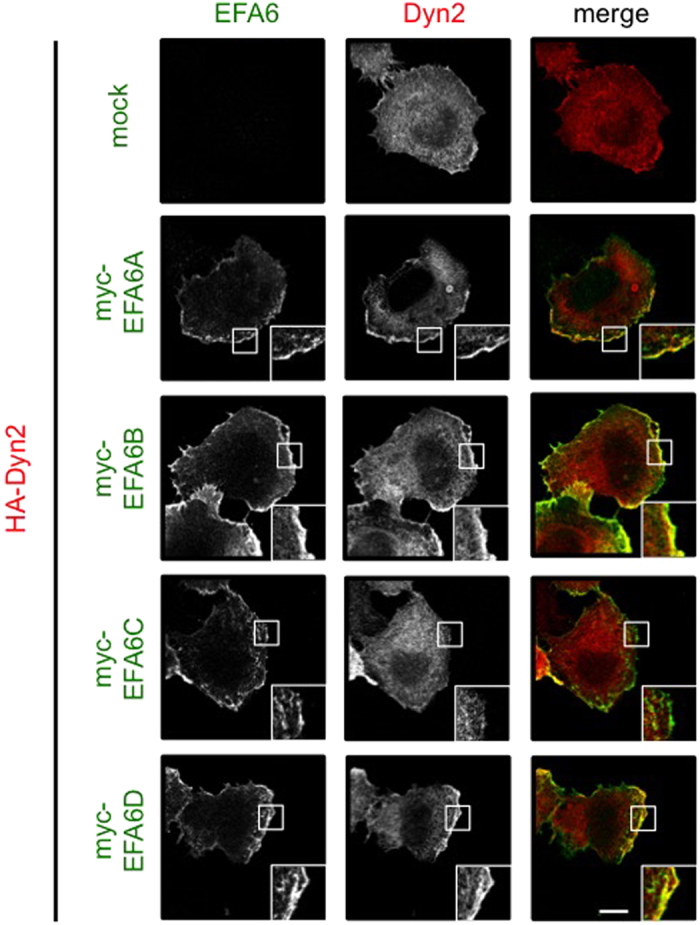
EFA6A, EFA6B and EFA6D colocalize with Dyn2 at cell periphery in HeLa cells. Myc-EFA6A-D were coexpressed with HA-Dyn2 in HeLa cells. After 24 hr of expression, cells were fixed and immunostained for EFA6s and Dyn2 with anti-myc and -HA antibodies, respectively. Boxed areas of cells were magnified and shown in the bottom corners in pictures. Shown are the representatives of three independent experiments. Scale bar, 10 μm.

If EFA6B and EFA6D do indeed mediate Dyn2-induced Arf6 activation, they could colocalize with Dyn2 in the cell. To investigate this hypothesis, myc-EFA6s were individually coexpressed with HA-Dyn2 in HeLa cells, and their localization was immunocytochemically assessed (Fig. 4). Consistent with the interaction data shown in Fig. 2C, EFA6A colocalized with Dyn2 (Fig. 4), although the mutant of EFA6A did not inhibit the Dyn2-induced Arf6 activation (Fig. 3). As expected, both EFA6B and EFA6D colocalized with Dyn2 at the cell periphery, while colocalization of EFA6C with Dyn2 was very week, if any, supporting our hypothesis described above.

### Binding Site of EFA6B for Dyn2

**Figure 5:**
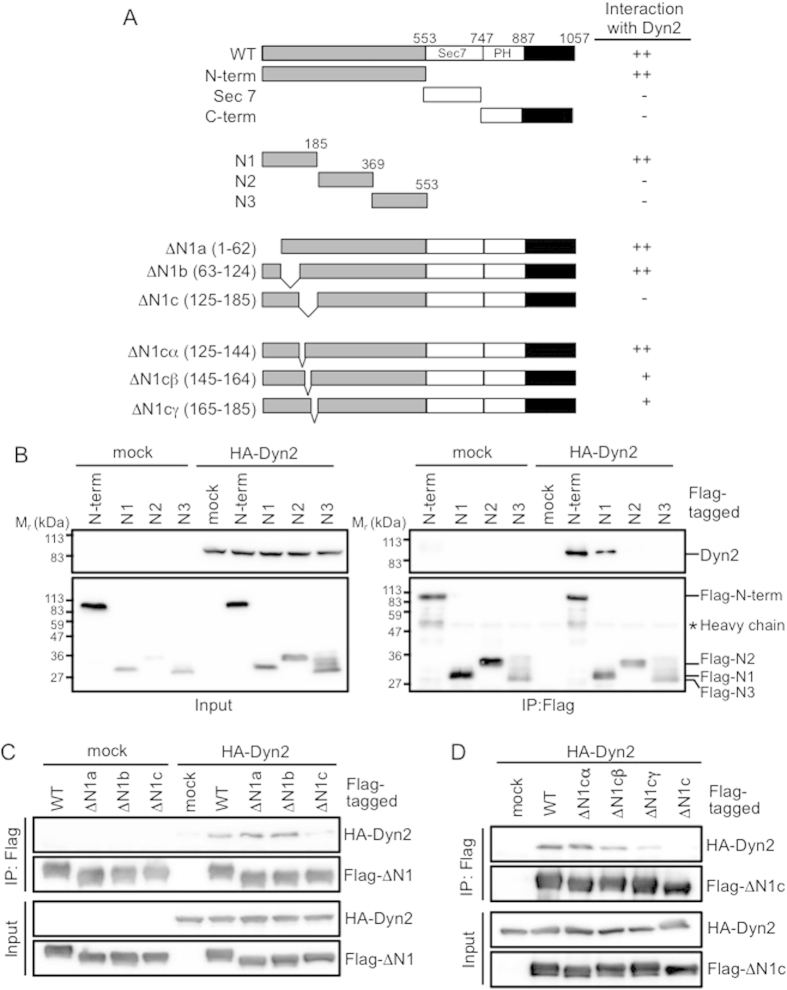
Identification of binding site of EFA6B for Dyn2. HA-Dyn2 and Flag-tagged deletion mutants of EFA6B were coexpressed in HEK293T cells. After immunoprecipitation of Flag-EFA6B mutants, coimmunoprecipitated Dyn2 was assessed by western blotting probed with anti HA-antibody.

A binding domain peptide of EFA6 for Dyn2 would be very useful for investigating the cellular functions of the Dyn2-EFA6-Arf6 signaling axis. To identify the binding site of EFA6B for Dyn2, a series of Flag-tagged deletion mutants of EFA6B illustrated in Fig. 5A were coexpressed with HA-Dyn2, and their interaction was assessed by an immunoprecipitation assay. The N-terminal domain of EFA6B, which does not contain any well-known motifs, interacted with Dyn2 to an extent comparable to the wild type of EFA6B, while Sec7 domain, which is responsible for GEF activity, and C-terminal domain including PH domain did not (Fig. 5A). Of the 3 N-terminus domains, N1, N2, and N3, which correspond to amino acids 1–185, 186–369 and 370–553, respectively, only N1 interacted with Dyn2 (Fig. 5A,B). Furthermore, ΔN1c mutant lacking amino acids 125–185 lost binding ability, while ΔN1a and ΔN1b mutants lacking the regions that correspond to amino acids 1–62 and 63–124, respectively, interacted with Dyn2 in a way comparable to the wild type of EFA6B (Fig. 5A,C). It was found that the mutants ΔN1cβ and ΔN1cγ, which delete the amino acids 145–164 and 165–185, respectively, became weaker in their ability to bind to Dyn2, while the mutant ΔN1cα retained its binding activity (Fig. 5A,D). Thus, the N-terminal 145–185 amino acid region of EFA6B seems to be critical for interaction with Dyn2, and its peptide could be useful to investigate the cellular functions of EFA6B.

### Dyn2-EFA6B/EFA6D-Arf6 Axis Is Involved in CME

**Figure 6:**
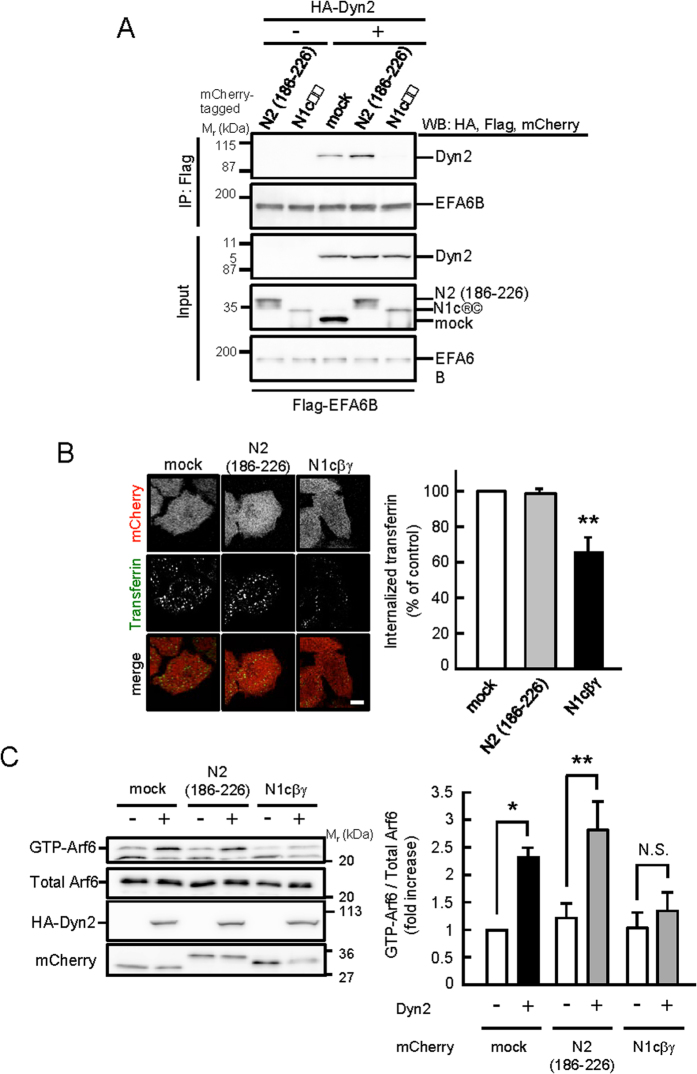
Involvement of Dyn2-EFA6B axis in transferrin uptake. (**A**) Flag-EFA6B and cDNA for the binding site peptide (mCherry-N1cβγ) or non-binding peptide of EFA6B (mCherry-N2 (186–226)) were coexpressed with HA-Dyn2 in HEK293T cells. After 48 hr of expression, peptides of EFA6B were immunoprecipitated with anti-Flag antibody beads, and coimmunoprecipitated Dyn2 was assessed by western blotting probed with anti-HA antibody. (**B**) HeLa cells transfected with mCherry-N1cβγ or mCherry-N2 (186–226) were cultured for 24 hr. After starvation for 30 min, cells were incubated with Alexa488-conjugated human transferrin at 37 °C for 10 min. The fluorescent intensity of internalized transferrin was measured as described in Methods. Right panels represent the mean ± SEM of percentages of internalized transferrin from three independent experiments. Statistical significance was calculated using Tukey multiple comparison test; **P < 0.01. Scale bar, 10 μm. (**C**) mCherry-N1cβγ or mCherry-N2 (186–226) was coexpressed with Arf6-Flag and with or without HA-Dyn2 in HEK293T cells. After 48 to 72 hr of expression, the levels of GTP-Arf6 were detected as in Fig. 1A (left panels). Right panel represents the mean ± SEM of the levels of GTP-Arf6 from six independent experiments. Statistical significance was calculated using Tukey multiple comparison test; *P < 0.05, **P < 0.01 and N.S., not significant.

Finally, we examined whether CME is regulated by the signaling pathway of Dyn2-induced Arf6 activation through EFA6B/EFA6D. We utilized the peptide N1cβγ of EFA6B (amino acids 145–185) that could bind to Dyn2 and inhibit the interaction between endogenous EFA6B and Dyn2. Overexpression of the mCherry-tagged N1cβγ peptide effectively inhibited the interaction between EFA6B and Dyn2 (Fig. 6A) and transferrin uptake (Fig. 6B), while the non-binding peptide N2 of EFA6B (amino acids 186–226) did not (Fig. 6A,B). Moreover, Dyn2-induced Arf6 activation was markedly inhibited by overexpression of N1cβγ but not N2 (Fig. 6C) These results support the notion that Dyn2-EFA6B/EFA6D-Arf6 axis is involved in CME, although colocalization of Dyn2 with EFA6B at the cell periphery was not affected by N1cβγ with unknown reason (data not shown).

This notion was also supported by the results obtained by knockdown of EFA6B, EFA6D, and Arf6 in HeLa cells. Consistent with the report that EFA6 is implicated in endocytosis^24^, transferrin uptake was suppressed by knockdown of either EFA6B or EFA6D (Supplementary Fig. S3A,B). Simultaneous knockdown of both EFA6B and EFA6D also inhibited the transferrin uptake to an extent comparable to that of the single knockdown of either of these EFA6s (Supplementary Fig. S3C). Furthermore, almost the same extent of reduction in the transferrin uptake by EFA6 knockdown was observed in the presence of the recycling inhibitor primaquine^25^ (Supplementary Fig. S3D), indicating that the reduction of transferrin uptake in EFA6 knockdown cells is most likely due to the defect in the endocytosis, but not accelerated recycling. Since fluid-phase endocytosis, which was assessed by the uptake of dextran, was not affected by double knockdown of EFA6B and EFA6D (Supplementary Fig. S4), EFA6s are involved only in CME. The transferrin uptake was also suppressed by Arf6 knockdown in the presence of primaquine (Supplementary Fig. S5). Furthermore, partial colocalization among Dyn2, EFA6, Arf6 and clathrin were observed at the cell periphery (Supplementary Fig. S6).

Thus, the Dyn2-EFA6B/EFA6D-Arf6 signaling axis, at least in part, regulates CME. However, the inhibition of transferrin uptake by these manipulations were only partial, suggesting that another signaling pathway is also involved in CME.

## Discussion

**Figure 7:**
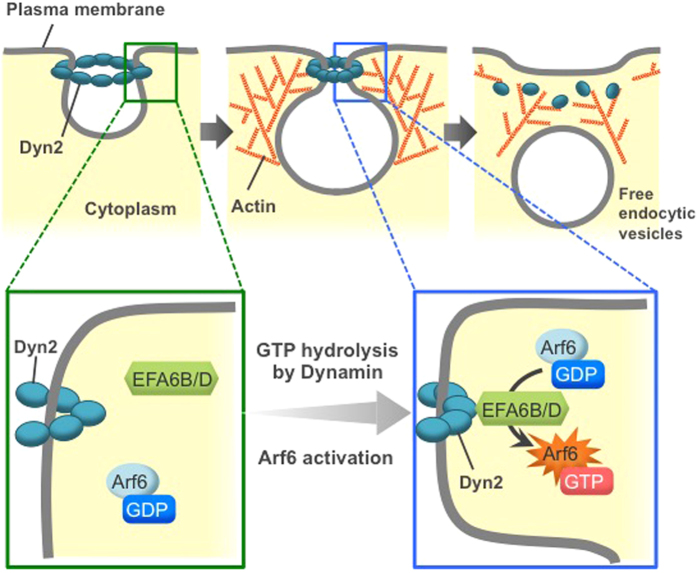
The proposed model for novel activation mechanism of Arf6 in CME. Dynamin2 (Dyn2) is recruited to the neck of the forming vesicles, where it induces membrane scission to produce free endocytic vesicles upon GTP hydrolysis. At this step, Dyn2 activates Arf6 through EFA6B and/or EFA6D.

In this study, we provide evidence for a novel activation mechanism of Arf6. Our results demonstrate that Dyn2, in a manner dependent upon GTPase activity, functions as an activator of Arf6 through the Arf6-specific GEFs, EFA6B and EFA6D, in CME (Fig. 7), consistent with the report that EFA6 is implicated in the regulation of endocytosis^24^.

The question arises as to the possible downstream effector(s) of Arf6 activated by Dyn2 in CME. Arf6 directly interacts with and activates the lipid-metabolizing enzymes, phospholipase D (PLD)^26^,^27^ and phosphatidylinositol 4-phosphate 5-kinase (PIP5K)^28^, which produce the versatile signaling lipids phosphatidic acid (PA) and phosphatidylinositol 4,5-bisphosphate (PI4,5P_2_), respectively. PI4,5P_2_ reorganizes the actin cytoskeleton through the regulation of actin-binding proteins such as the actin severing and capping protein gelsolin^29^,^30^,^31^. Based on these reports, it is reasonable to speculate that Arf6 reorganizes the actin cytoskeleton through the activation of PIP5K to coordinate with Dyn2 in the scission of the invaginated membrane. An alternative downstream molecule is PLD. Its product, PA, has been reported to facilitate endocytosis by forming the membrane curvature at the neck of deeply invaginated membrane^32^,^33^. It has also been reported that Dyn2 recruited to the neck of the invaginated membrane facilitates membrane curvature^34^. These reports led us to speculate that PLD functions as an alternative downstream effector to facilitate membrane curvature, which may be required for the scission of the invaginated membrane. In addition to these possible functions of PIP5K and PLD, these two lipid-metabolizing enzymes mutually accelerate their activation by a positive feedback mechanism. The PLD product PA is necessary for the activation of PIP5K by Arf6^28^, and the PIP5K product PI4,5P_2_ supports PLD activation by Arf6^35^.

We demonstrated that both EFA6B and EFA6D mediate Dyn2-induced Arf6 activation in CME. Another question raised here is how these two Arf6 GEFs share their functions in Dyn2-induced Arf6 activation in CME. It has been reported that Arf6 activation by EFA6 induces extensive membrane invagination as well as actin cytoskeletal reorganization in CME^24^. This report indicates that Arf6 plays multiple roles in several steps of the process of CME. Should this be the case, it is plausible that each EFA6 isozyme activate Arf6 sequentially at several steps in CME. This hypothesis would neatly explain why each dominant negative mutant of EFA6B and EFA6D significantly inhibited the Dyn2-induced Arf6 activation as shown in Fig. 3 and why each siRNA against EFA6B and EFA6D suppressed transferrin uptake to almost the same extent as shown in Supplementary Figure S3.

The results obtained in this study suggest that Arf6 activation is crucial in CME. However, ectopic expression of a constitutive active (Arf6 Q67L) as well as a dominant negative form of Arf6 (Arf6 T27N) inhibits transferrin internalization (Supplementary Fig. S7), consistent with the report that overexpression of EFA6 inhibits transferrin uptake^24^. These observation and report, taken together, imply that hyper-activation of Arf6 could disrupt CME and the appropriate cycling between active and inactive states of Arf6 is necessary for accomplishment of the entire process of CME. This idea is strongly supported by the report that Arf GAP SMAP1 regulates CME^36^.

Arf6 plays crucial roles in a wide variety of cellular events such as clathrin-dependent and -independent endocytosis, exocytosis, phagocytosis, macropinocytosis, and membrane ruffle and invadopodia formation via reorganization of actin cytoskeleton^10^. Dynamin has also been reported to regulate actin cytoskeletal reorganization-based cellular events, such as vesicle trafficking^37^, phagocytosis^38^, and invadopodia^39^, which are also regulated by Arf6. The similarities between dynamin and Arf6 in their cellular functions suggest that the Dyn2-EFA6-Arf6 axis plays important roles in a wide variety of actin cytoskeletal reorganization-related cellular events in addition to CME. It is of interest to investigate this further.

## Methods

### Reagents and Antibodies

Company-purchased chemicals and antibodies used in this study were as follows: Chemi-Lumi One from Nacalai Tesque, OPTI-MEM from GIBCO (Life Technologies), Glutathione Sepharose 4B from GE Healthcare, Lipofectamine 2000 from Invitrogen, anti-myc rabbit polyclonal antibody from MBL, anti-Flag M2 monoclonal antibody for western blotting and anti-Flag rabbit polyclonal antibody for immunostaining from SIGMA, anti-HA high affinity rat monoclonal antibody (clone 3F10) from Roche, anti-RFP rabbit polyclonal antibody from MBL, anti-clathrin mouse monoclonal antibody from Abcam, and horseradish peroxidase (HRP)-linked anti-mouse and anti-rat IgG antibodies from Cell Signaling Technology. Rabbit polyclonal antibody specific to Arf6 was generated as previously described^40^.

### Plasmids

cDNAs encoding Dyn2 and its mutant K44A, cytohesins, EFA6s, and BRAG2 were generous gifts from Dr. Nakayama (Kyoto University). These cDNAs were cloned into pcDNA3-Flag, pcDNA3-Myc, and pcDNA3-HA mammalian expression vectors, which were constructed by insertion of the epitope-encoding linkers into pcDNA3 (Invitrogen). Expression vectors for GEF-inactive mutants of EFA6s were constructed by introducing the mutations by PCR-based site-directed mutagenesis. cDNAs for deletion mutants of EFA6B were prepared by PCR using the pcDNA3 vector harboring full-length EFA6B as a template, and cloned into pcDNA3-Flag and pmCherry-C1 mammalian expression vectors (Clontech). All constructs were sequenced to confirm their identity.

### Cell Culture and Transfection

HeLa and HEK293T cells were cultured in DMEM containing 10% (v/v) fetal bovine serum (FBS), 50 units/ml penicillin and 50 units/ml streptomycin under 5% CO_2_. Cells were transiently transfected with plasmid DNAs or siRNAs by Lipofectamine 2000 according to the manufacturer’s protocol. After 4 hr of transfection, medium was changed to DMEM containing 10% FBS and cells were used for analyses.

### Assay of Arf6 Activation

HeLa cells were cotransfected with plasmids for either a wild type or a GTPase-deficient mutant of HA-Dyn2 and Arf6-Flag. In the experiment of Fig. 3, dominant negative mutants of EFA6A, EFA6B and EFA6D were also transfected. In the experiments with the inhibitor dynasore, transfected cells were treated with the dynasore as indicated in the figure legends. In the experiment for EFA6 knockdown, 40 nM siRNA against EFA6B or EFA6D (Dharmacon) were transfected with the plasmid for Arf6-Flag and with or without the plasmid for HA-Dyn2. These cells were lysed in a buffer consisting of 150 mM NaCl, 25 mM Tris-HCl, pH 7.2, 5 mM MgCl_2_, 1% NP-40, 5% glycerol, 10 μg/ml aprotinin, 1 μg/ml leupeptin, and 1 μg/ml pepstatin, and incubated on ice for 5 min. After centrifugation at 16,000 × g for 15 min at 4 °C, the GTP-bound active form of Arf6 in the supernatant was pulled down with glutathione-Sepharose beads (GE Healthcare) conjugated with glutathione *S*-transferase (GST)-tagged leucine zipper region II (LZII) (amino acids 398–455) of JNK-interacting protein (JIP), which specifically binds to the active form of Arf6^41^. After the beads were washed with lysis buffer 3 times and suspended in the SDS sample buffer, proteins were separated by SDS-PAGE and the active form of Arf6 was analyzed by western blotting with anti-Flag antibody as described previously^42^.

### Interaction of Dyn2 with Arf6 GEFs and Identification of Binding Site of EFA6B for Dyn2

To investigate the interaction of Dyn2 with Arf6 GEFs, HEK293T cells were coexpressed with HA-Dyn2 and Flag-Arf6 GEFs. To identify the binding site of EFA6B for Dyn2, HEK293T cells were coexpressed with HA-Dyn2 and Flag-tagged deletion mutants of EFA6B. Cells were then solubilized in a lysis buffer (25 mM Tris-HCl, pH7.5, 10 mM KCl, 1% Triton-X, 1 mM EDTA, 0.1 mM EGTA, 5 mM MgCl_2_, 10% Glycerol, 5 mM NaF, 4 mM Na_3_VO_4_, 0.8 mM Na_2_P_2_O_7_, 1 mM PMSF, 2 μg/ml aprotinin, 2 μg/ml leupeptin, 1 μg/ml pepstatin). Flag-Arf6 GEFs and Flag-EFA6B mutants were immunoprecipitated with anti-Flag M2 affinity gel (SIGMA-ALDRICH). Proteins in the immunoprecipitates were blotted with anti-HA and -Flag antibodies.

### Localization of Dyn2 and EFA6s in HeLa Cells

HA-Dyn2 and myc-EFA6s were coexpressed in HeLa cells at 37 °C for 24 hr. After cells were fixed with 4% paraformaldehyde in ice-cold PBS, Dyn2 and EFA6s were stained with anti-HA and anti-myc antibodies, respectively. Images were obtained with a laser-scanning confocal microscope (TCS SP5; Leica Microsystems).

### Assays for Transferrin and Dextran Uptake

HeLa cells transfected with the td-Tomato expression plasmid and siRNAs for EFA6B and/or EFA6D (Invitrogen) were cultured in DMEM supplemented with 10% FBS for 24 hr. In the experiment with the binding peptide, HeLa cells were transfected with cDNA for mCherry-N2 (186–226 amino acids) or -N1cβγ peptide of EFA6B, followed by incubation in DMEM supplemented with 10% FBS for 24 hr. After starvation in DMEM with 0.1% BSA for 30 min, cells were incubated with 25 μg/ml of Alexa488- or Alexa594-conjugated human transferrin or 0.1 mg/ml of Alexa-488 conjugated dextran at 37 °C for indicated times with or without 3 μM of primaquine (SIGMA). They were rinsed with ice-cold PBS, washed with acid stripping solution consisting of 50 mM NaCl, 2 mM CaCl_2_, and 25 mM CH_3_COONa, pH4.5, at 4 °C for 5 min, and fixed with 4% paraformaldehyde in ice-cold PBS. Images of the cells were captured with a laser-scanning confocal microscope (TCS SP5; Leica Microsystems). The fluorescent intensity of internalized transferrin or dextran was measured by image J software. In short, td-Tomato, mCherry-expressed or Arf6-GFP expressed cells were outlined to measure cell area. The intensity of 488- or 594-transferrin or 488-dextran was normalized by cell area, and averaged over individual cells.

## Additional Information

**How to cite this article**: Okada, R. *et al.* Activation of the Small G Protein Arf6 by Dynamin2 through Guanine Nucleotide Exchange Factors in Endocytosis. *Sci. Rep.* **5**, 14919; doi: 10.1038/srep14919 (2015).

## Supplementary Material

Supplementary Information
