# Supplementary material for: Activation of the Small G Protein Arf6 by Dynamin2 through Guanine Nucleotide Exchange Factors in Endocytosis
Source: Sci Rep. 2015 Oct 27;5:14919. doi: 10.1038/srep14919 (PMC4621509; doi:10.1038/srep14919)
Supplement: Supplementary Information [file srep14919-s1.pdf]

Supplementary Figures

Activation of the Small G Protein Arf6 by Dynamin2 through Guanine Nucleotide Exchange Factors in Endocytosis

Risa Okada, Yohei Yamauchi, Tsunaki Hongu, Yuji Funakoshi, Norihiko Ohbayashi, Hiroshi Hasegawa, Yasunori Kanaho

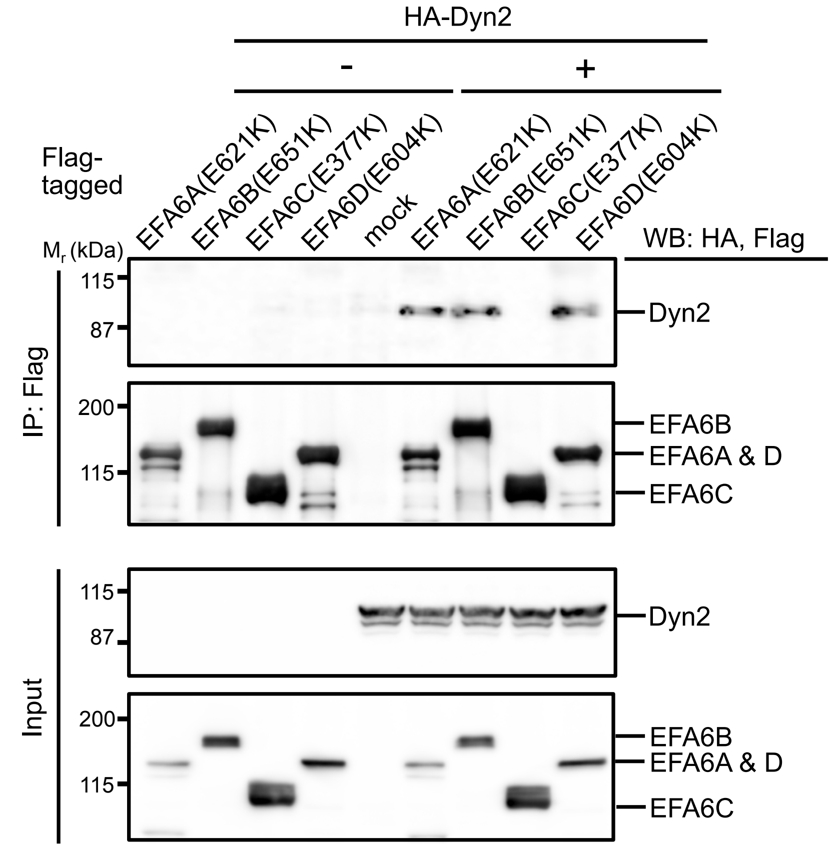

Supplementary Figure S1. Interaction of EFA6 mutants with Dyn2. Flag-tagged dominant negative EFA6 mutants were individually coexpressed with HA-Dyn2 in HEK293T cells. After 48 hr of expression, EFA6s were immunoprecipitated with anti-Flag antibody beads, and coimmunoprecipitated Dyn2 was assessed by western blotting with anti-HA antibody.

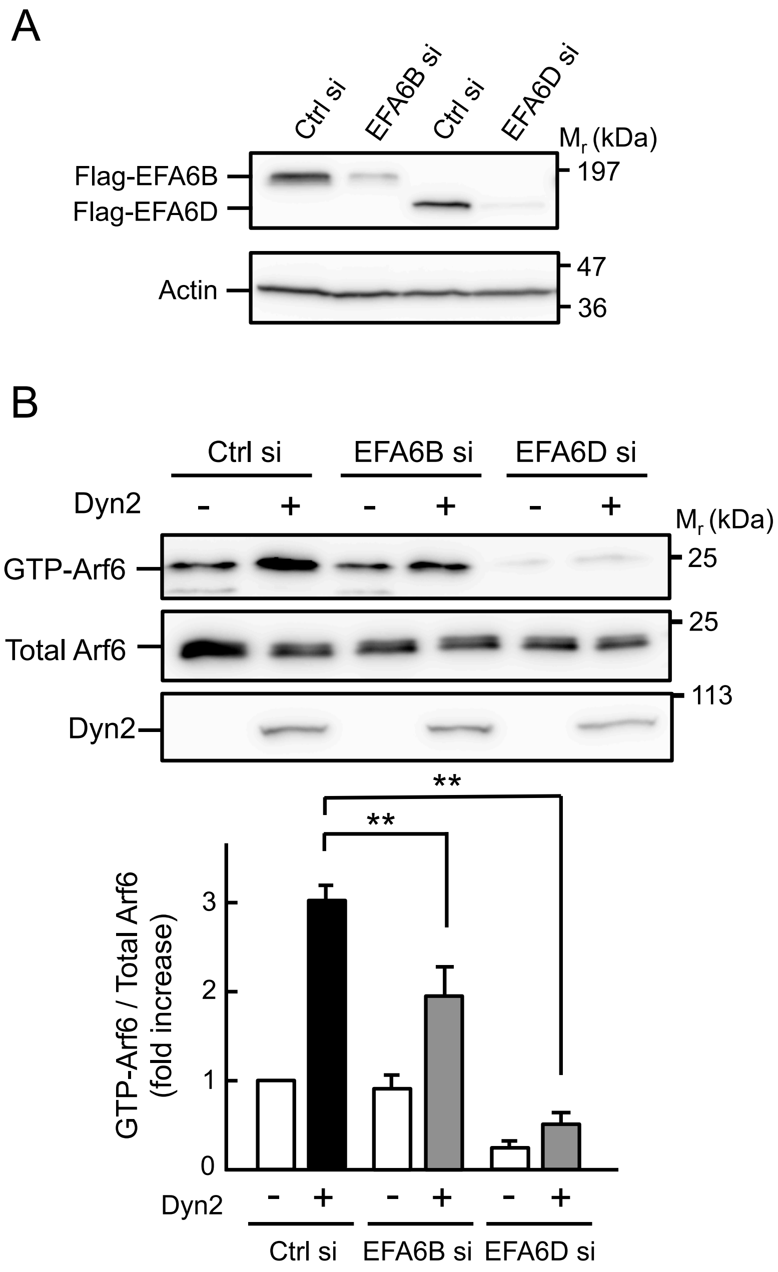

Supplementary Figure S2. Effects of EFA6 knockdown on Dyn2-induced Arf6 activation. (A) HEK293T cells were cotransfected with the plasmid and siRNA for EFA6B or for EFA6D. Knockdown efficiencies of these siRNAs were assessed by western blotting 24 hr after transfection. (B) The plasmid for Arf6-Flag and either siRNA against EFA6B or EFA6D were cotransfected with or without the plasmid for HA-Dyn2 in HEK293T cells. After 48 to 72 hr of expression, the levels of GTP-Arf6 were detected as in Fig. 1A (upper panels). Lower panel represents the mean ± SEM of the levels of GTP-Arf6 from five independent experiments. Statistical significance was calculated using Tukey multiple comparison test; $^{**}P<0.01$.

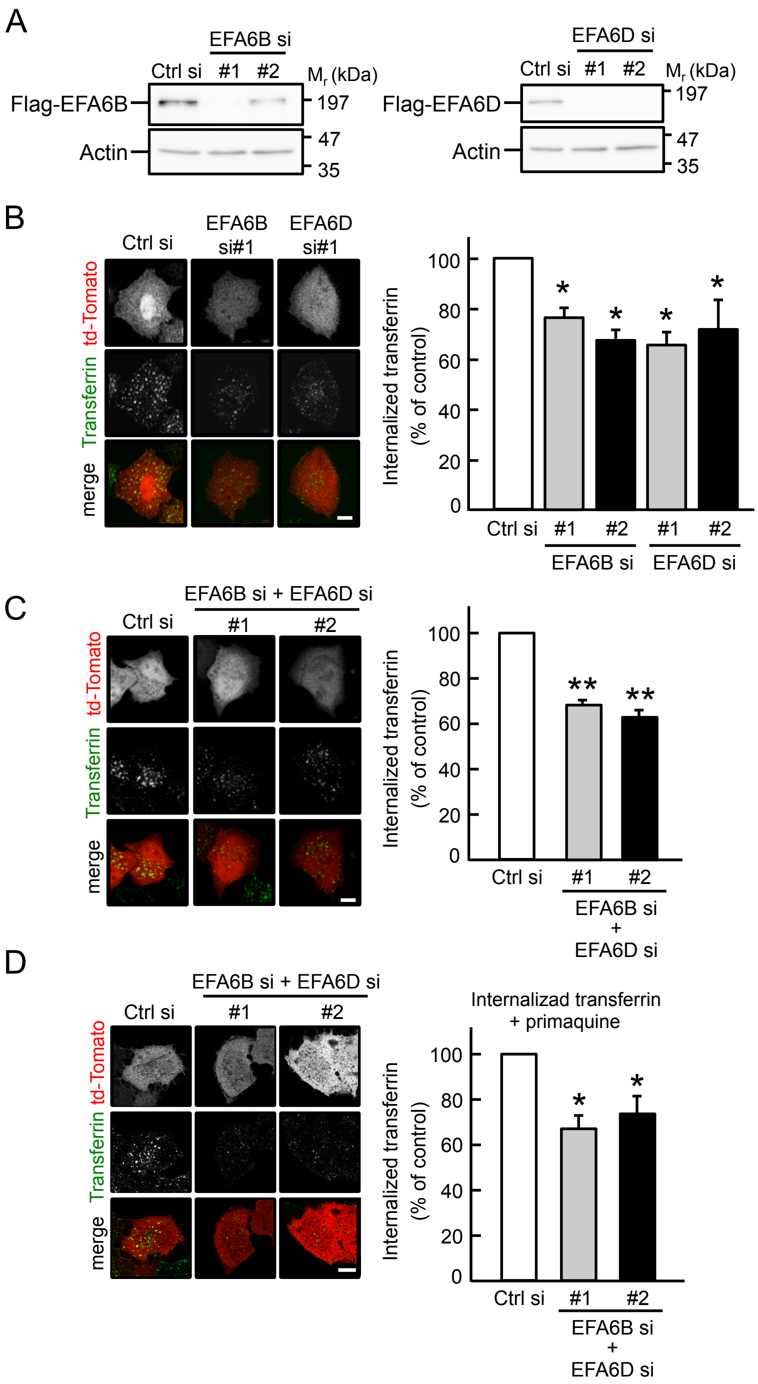

Supplementary Figure S3. Involvement of EFA6B and EFA6D in transferrin uptake. (A) HeLa cells were transfected with the plasmid and siRNAs for Flag-EFA6B and EFA6B, respectively, or for Flag-EFA6D and EFA6D, respectively. Knockdown efficiencies of these siRNAs were assessed by western blotting 24 hr after transfection. (B-D) HeLa cells transfected with td-Tomato-expression plasmid and siRNAs against EFA6B or EFA6D (B) or both siRNAs against EFA6B and EFA6D (C, D) were cultured for 24 hr. After starvation for 30 min, cells were incubated with Alexa488-conjugated human transferrin in the absence (B, C) or presence of 3 μM of primaquine (D) at 37°C for 10 min. The fluorescent intensity of internalized transferrin was measured as described in Methods. Right panels represent the mean ± SEM of percentages of internalized transferrin from three independent experiments. Statistical significance was calculated using Tukey multiple comparison test; *P<0.05 and **P<0.01. Scale bar, 10 μm.

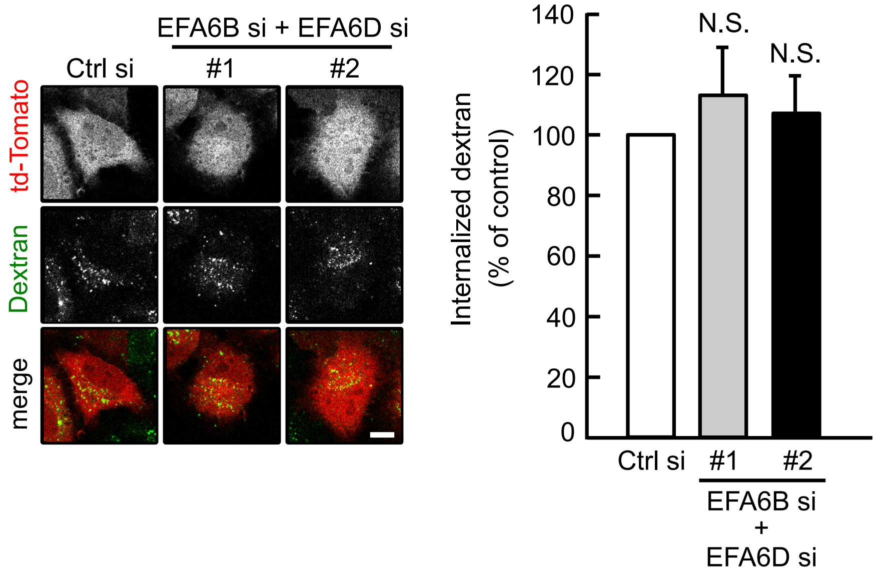

Supplementary Figure S4. Fluid-phase endocytosis was not affected by double knockdown of EFA6B and EFA6D. HeLa cells transfected with the plasmid for td-Tomato and both siRNAs against EFA6B and EFA6D were cultured for 24 hr. Cells were incubated with Alexa488-conjugated dextran in the presence of 3 μM of primaquine at 37°C for 30 min. The fluorescent intensity of internalized dextran was measured as described in Methods. Right panels represent the mean ± SEM of percentages of internalized dextran from four independent experiments. Statistical significance was calculated using Tukey multiple comparison test; N.S. not significant. Scale bar, 10 μm.

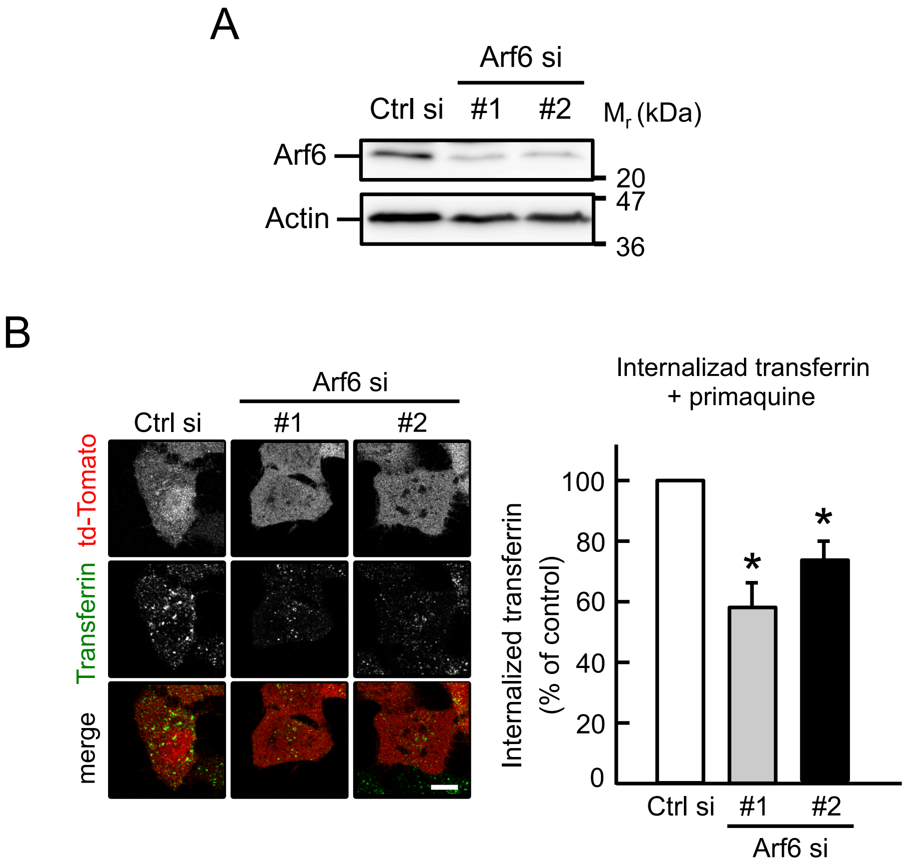

Supplementary Figure S5. Transferrin uptake was suppressed by knockdown of Arf6. (A) Lysates prepared from HeLa cells transfected with siRNAs for control and Arf6 were western blotted with anti-Arf6 antibody and anti-actin antibody. (B) HeLa cells transfected with a td-Tomato-expression plasmid and siRNAs against Arf6 were cultured for 24 hr. The cells were starved for 30 min, then incubated with Alexa488-conjugated human transferrin in the presence of 3 μM of primaquine at 37°C for 10 min. The fluorescent intensity of internalized transferrin was measured as described in Methods. Right panels represent the mean ± SEM of percentages of internalized transferrin from four independent experiments. Statistical significance was calculated using Tukey multiple comparison test; *P<0.05. Scale bar, 10 μm.

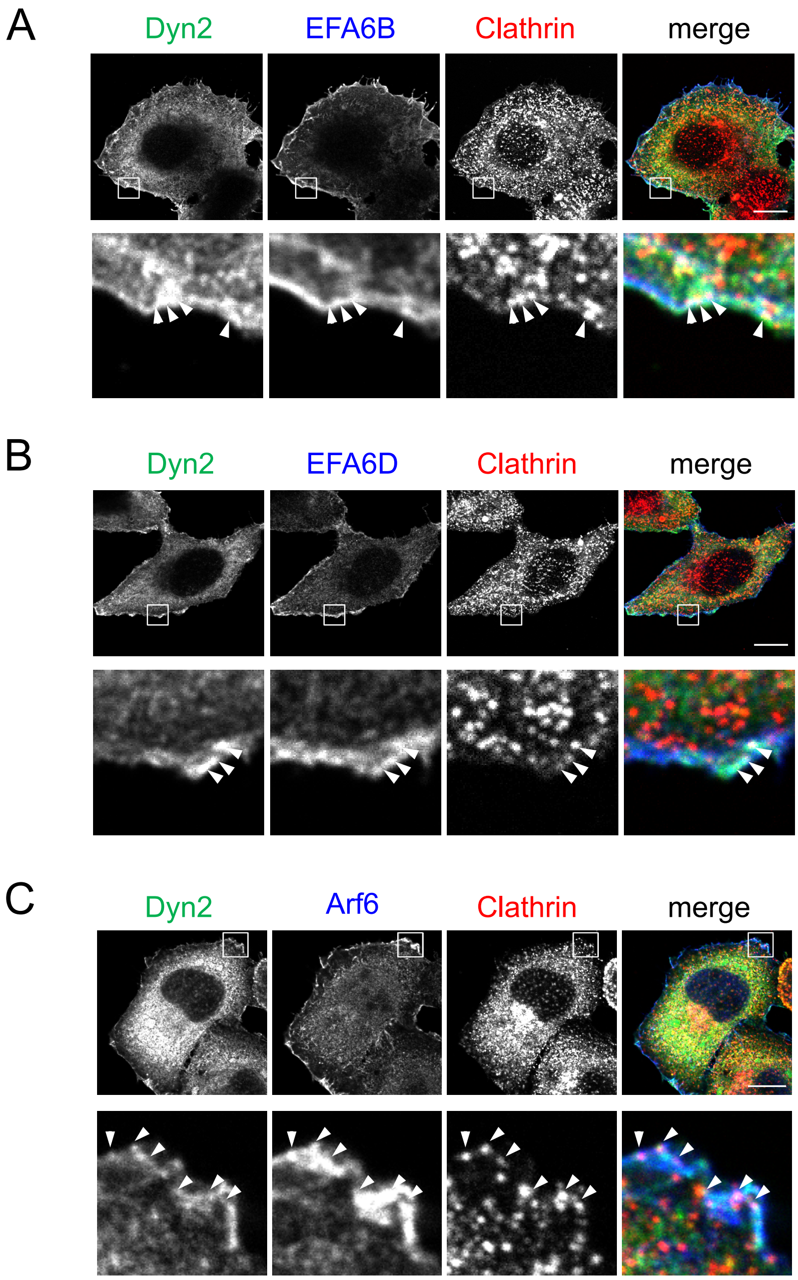

Supplementary Figure S6. Colocalization of clathrin with Dyn2, EFA6 and Arf6. Myc-EFA6B (A), myc-EFA6D (B), or Arf6-Flag (C) were coexpressed with HA-Dyn2 in HeLa cells. After 24 hr of expression, cells are fixed and immunostained with anti-clathrin, anti-HA and anti-myc (A, B) or anti-Flag antibodies (C). Boxed areas of cells were magnified in the lower panels. White arrowheads, triple-positive signals. Scale bar, 10 μm.

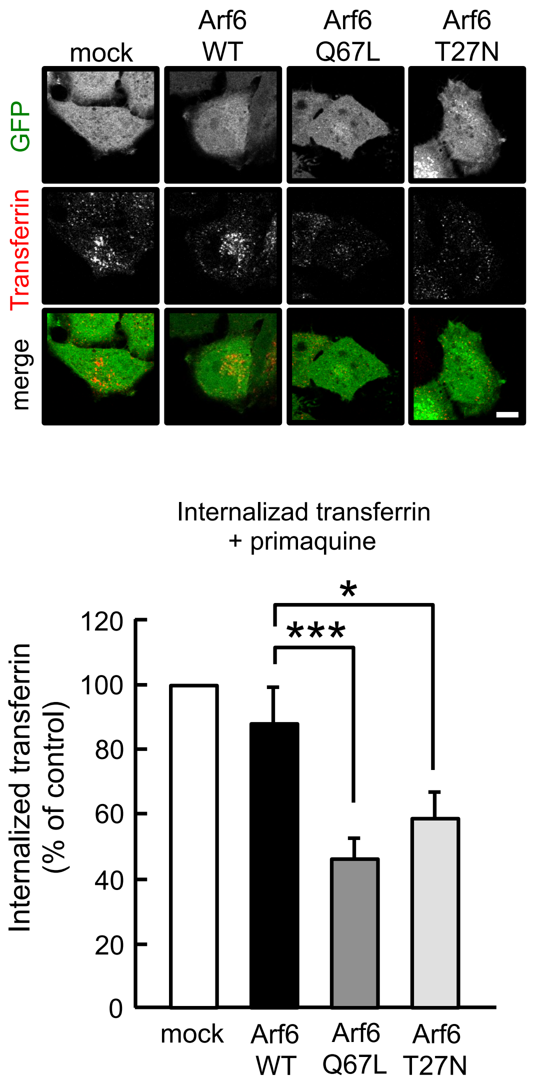

Supplementary Figure S7. Overexpression of constitutively active and dominant negative form of Arf6 inhibits transferrin uptake. HeLa cells transfected with GFP-tagged wild type (Arf6 WT-GFP), constitutively active (Arf6 Q67L-GFP) or dominant negative form of Arf6 (Arf6 T27N-GFP) were cultured for 24 hr. After starvation for 30 min, cells were incubated with Alexa594-conjugated human transferrin in the presence of 3 μM of primaquine at 37°C for 10 min. The fluorescent intensity of internalized transferrin was measured as described in Methods. Lower panels represent the mean ± SEM of percentages of internalized transferrin from six independent experiments. Statistical significance was calculated using Tukey multiple comparison test; *P<0.05 and ***P<0.005. Scale bar, 10 μm.
